# Chloramine T in pikeperch (*Sander lucioperca*) aquaculture. Safeguard or hidden threat? Evaluating its effect on fish growth, immune response and liver function

**DOI:** 10.2478/jvetres-2026-0006

**Published:** 2026-02-12

**Authors:** Patrycja Schulz, Elżbieta Terech-Majewska, Maciej Rożyński, Karolina Duk, Joanna Pajdak-Czaus, Andrzej Krzysztof Siwicki, Zdzisław Zakęś

**Affiliations:** Department of Ichthyopathology and Fish Health Prevention, National Inland Fisheries Research Institute, 05-500 Piaseczno, Poland; Department of Infectious and Invasive Diseases and Veterinary Administration, Institute of Veterinary Medicine, Faculty of Biological and Veterinary Sciences, Nicolaus Copernicus University in Toruń, 87-100 Toruń, Poland; Department of Aquaculture, National Inland Fisheries Research Institute, 10-719 Olsztyn, Poland; Veterinary Diagnostic Laboratory ALAB Plus – ALAB bioscience, 00-739 Warsaw, Poland; Department of Epizootiology, Faculty of Veterinary Medicine, University of Warmia and Mazury in Olsztyn, 10-719 Olsztyn, Poland

**Keywords:** aquaculture health management, disinfectants, fish welfare and productivity, hepatotoxicity, recirculating aquaculture system

## Abstract

**Introduction:**

Chloramine T (CLT) is utilised in aquaculture for its disinfectant and antiseptic properties, yet its physiological effects on fish health remain underexplored, particularly in emerging species like pikeperch (*Sander lucioperca*). This study assessed the impact of CLT exposure on growth performance, immune response and liver function in pikeperch reared in recirculating aquaculture systems (RAS).

**Material and Methods:**

A total of 270 fish were assigned to a control group (C), a single-bath group (10 mg L^−1^, day 0; CLT1) and a three-bath group (10 mg L^−1^ on days 0, 7 and 14; CLT3). Fish were reared in independent RAS units under stable physicochemical conditions. Growth performance was monitored, and blood and tissue were sampled. Liver enzyme (ALT, AST and alkaline phosphatase), serum lysozyme and ceruloplasmin activities, phagocyte function, lymphocyte proliferation and liver histopathology were assessed.

**Results:**

Growth parameters and condition factor (ratio of body weight to the cube of body length) remained stable across all groups, and no mortality was observed. Liver enzyme activities showed no significant alterations; however, inflammatory changes were noted in the liver in the CLT3 group. Serum lysozyme activity increased after the initial exposure, indicating an acute immune response. In contrast, repeated exposure reduced respiratory burst and phagocyte killing activity, suggesting immunosuppression; however, CLT3-group T and B lymphocytes proliferated more as a reaction to inflammation.

**Conclusion:**

While CLT at 10 mg L^−1^ is indicated to be generally safe for pikeperch under controlled RAS conditions, repeated exposures may induce mild hepatic inflammation and temporary immune modulation. This study offers insights into optimising the use of CLT in aquaculture to ensure efficacy and safety.

## Introduction

Fish health is a critical factor in the sustainable development of aquaculture, directly impacting the industry’s economic viability (24). In recent decades, the rapid expansion of aquaculture to meet global demand for fish products has led to intensified production. While this can result in increased harvests, it also creates conditions, such as high stocking densities, stressful rearing environments and altered water quality, that predispose fish to stress and disease. Traditionally, antibiotics have been essential tools for disease prevention and treatment. However, their overuse and misuse have raised significant concerns owing to the emergence of antibiotic-resistant bacteria in the environment. Consequently, protecting fish farms and supporting sustainable practices requires a shift toward alternative therapies that effectively maintain fish health without contributing to resistance (9).

One of the most effective approaches to minimising disease outbreaks in aquaculture is the implementation of strict biosecurity measures. Biosecurity encompasses a range of preventive strategies aimed at reducing or completely preventing the introduction and spread of infectious agents. These include hygiene protocols, health monitoring, proper treatment interventions and disinfection and antisepsis (18). Among these measures, prophylactic and therapeutic baths have proved to be viable methods for controlling pathogen loads. By disinfecting the aquatic environment and maintaining the asepsis of fish skin, infection risk is reduced and overall system biosecurity enhanced (14). In this context, disinfection involves eliminating both vegetative and spore forms of harmful microorganisms transmitted through various vectors and preventing their re-establishment on equipment and surfaces. Complementing this, antiseptics are used to reduce or eradicate microorganisms on living tissues such as gills, skin and mucous membranes. In aquaculture, these are commonly applied *via* water-based treatments during egg incubation, larval rearing and juvenile growth and as part of broodstock maintenance. Many germicides are used for both purposes, but maximum concentrations used for antisepsis are lower than for disinfection to prevent damage to living tissue (18).

Among promising antimicrobial agents, chloramine T (CLT), N-chloro-p-toluenesulfonamide sodium salt, has gained attention for its dual antiseptic and disinfectant properties. Widely used in human and veterinary medicine and water sanitation, CLT is known for its potent biocidal activity. Upon dissolution in water, CLT hydrolyses to release hypochlorite ions (OCl^–^), which form hypochlorous acid (HOCl) – a powerful agent capable of disrupting microbial cell structures and inactivating pathogens rapidly. Its broad-spectrum antimicrobial activity, high solubility, biodegradability and low bioaccumulation potential make it a strong candidate for use in aquaculture (32). Research has demonstrated CLT’s efficacy against a wide spectrum of pathogens, including viruses, bacteria, water moulds and parasites such as *Flavobacterium psychrophilum, F. branchiophilum* (18), *Aeromonas salmonicida, Vibrio anguillarum, V. salmonicida, Yersinia ruckerii* and infectious pancreatic necrosis virus (8). Moreover, CLT has been approved by the U.S. Food and Drug Administration as a preparation for reducing mortality caused by bacterial gill disease in freshwater-reared salmonids and by columnaris in warmwater finfish (32).

As interest increases in the use of CLT in aquaculture, it is essential to evaluate its safety and physiological effects across different fish species. Each species possesses distinct physiological and immunological characteristics that significantly influence its tolerance of chemical treatments. While some species can tolerate certain disinfectants without adverse effects, others may experience considerable stress, immunosuppression or organ dysfunction. This highlights the critical need to assess the sensitivity of species newly introduced into intensive aquaculture. Pikeperch (*Sander lucioperca*), an emerging species in European aquaculture and one in growing demand in the market on account of its high-quality meat (19), represents one such fish. Scientific data on its tolerance to chemical agents such as CLT remain scarce. In the absence of such information, the use of chemical treatments in its culture could lead to unintended consequences, including impaired fish health and reduced production performance. To address this knowledge gap, the aim of the present study was to evaluate the effects of both single and repeated CLT exposures on growth performance, immune response and liver function in pikeperch reared in recirculating aquaculture systems (RAS). These parameters are key indicators of fish health and are essential for determining the species’ suitability for intensive aquaculture production.

## Material and Methods

### Experimental set-up

The material was obtained using the out-of-season spawning and rearing method for pikeperch in RAS (34). The experiment involved fish with an average body weight (BW) of 121.7 ± 16.9 g and an average standard length (SL) of 21.3 ± 1.0 cm. Fish were divided into three groups in triplicate: C, control fish not bathed in chloramine; CLT1, fish bathed once in chloramine-T (on day 0); and CLT3, fish bathed three times in chloramine-T (on days 0, 7 and 14).

Each group was placed in a separate, independent RAS. Each system comprised three rearing tanks, each having a volume of 0.2 m^3^, and a fluidised bed biofilter filled with injection-moulded RK BioElements filter media (Dania Plast, Skive, Denmark) with a biological bed volume of 100 L. The total volume of each RAS, including the mechanical filter, biological bed, bottom tank, top tank, rearing pools and pipes, was 1,350 L. Thirty fish were placed in each tank, resulting in a total of 270 fish being used in the experiment. During rearing, the water temperature (± 0.1°C) and its oxygen concentration (± 0.01 mg O_2_ L^−1^) were measured daily at the inflow and outflow of the rearing tanks. The average water temperature was 21.8 ± 0.1°C and the oxygen concentration at the outflow from the rearing tanks did not fall below 7.5 mg O_2_ L^−1^. Other water parameters, *i.e*. concentration of total ammoniacal nitrogen (TAN = NH_4_^+^-N + NH_3_-N; ± 0.01 mg TAN L^−1^), nitrite (± 0.01 mg NO_2_-N L^−1^), and total hardness and pH were measured twice a week also at the inflow and outflow of the rearing tanks. Total ammoniacal nitrogen and NO_2_-N concentrations at the outflow did not exceed 0.17 mg TAN L^−1^ and 0.063 mg NO_2_-N L^−1^, respectively. Total hardness was 384.2 mg CaCO_3_ L^−1^, and pH was between 7.72 and 7.90. The water flow in the rearing tanks was 4 L min^−1^ (1.2 exchange h^−1^). During the study, the fish were fed with Aller Aqua Ivory feed (Aller Aqua, Christiansfeld, Denmark) with a protein content of 54.0%, carbohydrate content of 13.0%, crude fat content of 15.0%, crude fibre content of 1.5% and gross energy content of 21.1 MJ kg^−1^ (manufacturer’s data). Feed was given using automatic feeders (Fischtechnik, Nienburg, Germany) continuously for 19 h d^−1^ (10:00–05:00). The daily feed rate was 1.2% of the stocking biomass. No fish of any group were fed on days 0, 7 and 14. A photoperiod of 24L: 0D was used, and the light intensity measured at the water surface in the rearing tanks was 30–40 lux.

### Treatment application protocol

During the experiment, two procedures involving prophylactic CLT baths (100% CLT; Chempur, Piekary Śląskie, Poland) were tested. The CLT1 group was bathed once starting on day 0 for 24 h in CLT at a nominal concentration of 10 mg L^−1^. The CLT3 group was bathed three times starting on day 0, starting on day 7 and starting on day 14, also for 24 h each time. Before the addition of CLT to the RAS, faeces and uneaten feed were removed from the rearing pools, fresh tap water was turned off and any water losses in the RAS were made up. Next, 10 L of water was drawn from the top tank of the RAS, in which a precise dose of CLT (± 0.1 mg) was dissolved. This solution was poured back into the top tank. After the 24-h CLT bath, each RAS was topped up with fresh tap water at 0.16 L^−1^ over 24 h, and during this time the water was changed fully in the RAS. Following this initial top-up, the volumes of fresh water added were reduced to the standard technological volumes (2% of RAS volume d^−1^). Individual fish measurements of SL (± 0.1 cm) and BW (± 0.01 g) were taken before the first CLT bath on day 0 and then on days 20 and 36. The data collected allowed the calculation of the fish condition factor: F = 100 × BW × SL^−3^. In addition, fish mortality was monitored daily.

### Sample collection

Seven blood samples including blood from two or three individuals from each rearing tank were collected on days 1, 8 and 15 for biochemical tests. Three weeks after the last bath of the CLT3 group, all groups were sampled on day 36. Blood was collected from the tail vein using heparinised syringes (Smiths Medical, Minneapolis, MN, USA). For immunological and histopathological assays, samples were taken from six individuals from each group on day 1 after CLT exposure and then at weekly intervals. Blood was collected from the caudal vein and transferred to sterile tubes. The fish were individually captured and euthanised in a 100 mg L^−1^ aqueous solution of MS-222 anaesthetic (Sigma-Aldrich, St. Louis, MO, USA) (23). After the fish were euthanised, the spleen and pronephros of each fish were removed and examined immediately. During necropsy, a fragment of the liver lobe was collected, placed in a numbered histological cassette, assigned a random five-digit identification number and fixed for 24 h in Davidson’s solution (11).

### Liver biochemical marker analysis

Blood was centrifuged at 6,500 rpm for 3 min at 20°C (Fresco 17; Thermo Fisher Scientific, Waltham, MA, USA). Plasma was analysed using an automatic biochemical analyser (BS120; Mindray, Shenzhen, China) with reagent kits specific to this equipment. The following enzyme activities were determined: ALT (in a kinetic assay read at optical density (OD) of 340 nm; cat. No. 96.022), AST (also in a kinetic assay read at 340 nm; cat. No. 96.042), and alkaline phosphatase (ALP; in a kinetic assay read at 405 nm; cat. No. 96.130; all kits from Stamar, Dąbrowa Górnicza, Poland). Calibration and internal quality control were performed according to the manufacturer’s instructions. Results were reported as enzyme activity units.

### Immunological assays

The immunological analyses were carried out according to Siwicki *et al*. (28). After centrifugation of blood samples at 2,000 × *g* for 10 min at 4°C, the serum was collected and stored at –20°C until analyses were performed.

Lysozyme activity (LSM) was determined using a turbidimetric assay (Sigma-Aldrich) based on the lysis of *Micrococcus lysodeikticus*. Absorbance at 450 nm was measured immediately after bacterial addition and after 1 h of incubation at 25°C. Ceruloplasmin activity was assessed spectrophotometrically using p-phenylenediamine (Sigma-Aldrich) as a substrate, with OD recorded at 540 nm. Total protein levels were measured in the biuret reaction, and γ-globulin levels were determined by polyethylene glycol precipitation with OD also measured at 540 nm.

Immune cells were isolated from the spleen and pronephros by passing tissue through a 70-μm cell strainer and subsequently separating the cells on a density gradient using Gradisol L (Aqua-Medica, Łódź, Poland). Cells were suspended in RPMI-1640 medium containing 10% foetal calf serum and 1% antibiotic-antimycotic solution (both reagents from Sigma-Aldrich) and incubated in 96-well plates at 24°C for analysis. Phagocyte respiratory burst activity was measured by nitroblue tetrazolium (NBT, Sigma-Aldrich) reduction after stimulation with phorbol myristate acetate (Sigma-Aldrich). Phagocyte potential killing activity (PKA) was assessed using NBT reduction in the presence of *Aeromonas hydrophila*. After 30 min of incubation at 24°C, reduced NBT was solubilised, and OD at 620 nm was measured. All samples were tested in triplicate. The stimulation index (SI) was calculated by dividing the OD of stimulated cells by that of control cells.

Lymphocyte proliferation was evaluated using the 3-(4,5-dimethyl thiazol-2-yl)-2,5-diphenyltetrazolium bromide (MTT) assay after stimulation with concanavalin A (Sigma-Aldrich) for T cells and lipopolysaccharide (Sigma-Aldrich) for B cells. Cells were incubated at 22°C for 48 h, after which MTT was added for 3 h at 24°C. The reduced formazan product was dissolved in dimethyl sulphoxide, and the OD at 570 nm was recorded. All assays were performed in triplicate. The SI was calculated by dividing the OD of stimulated cells by that of the control cells.

### Histology

After 24 h fixation, the tissues were rinsed three times in a 70% ethanol solution and subjected to standard histopathological processing. They were initially dehydrated in a series of alcohols from the initial concentration of 70% using an automatic tissue processor (TP102; Leica Biosystems, Nussloch, Germany). After removal from the processor, the samples were embedded in paraffin blocks, and after freezing, they were cut on a rotary microtome (RM2255; Leica Biosystems) into 5-μm sections and applied to slides. Slides were stained with haematoxylin and eosin on a programmable stainer (ST5010 Autostainer XL; Leica Biosytems) according to Bancroft and Layton (5).

Prepared slides were analysed using a light microscope (Nikon Eclipse 80i; Tokyo, Japan). The histopathological assessment method for the liver followed the approach described by Bernet *et al*. (6). This semiquantitative scoring method involves assigning each observed pathological change to an appropriate reaction pattern and grading the change on a scale of 0 to 6, which represents the severity of the lesion and its distribution within the sample. Each alteration has an *a priori* assigned importance factor ranging from 1 to 3 based on its reversibility and long-term impact on organ function. Hepatic alterations are categorised into five reaction patterns, an index being calculated for each pattern as the product of the grading and the importance factor. The indices are circulatory disturbances (I_LC_), regressive changes (I_LR_), progressive changes (I_LP_), inflammatory responses (I_LI_) and neoplastic changes (I_LT_). The total organ index (I_L∑_) was calculated by adding up all reaction pattern indices.

### Statistical analysis

In the assessment of body weight, condition factor, liver enzyme activity and humoral immunity parameters, mean values and standard deviations were used for comparisons between groups. For the analysis of cellular immunity parameters, the SI was used. Statistica 12 (StatSoft, Tulsa, OK, USA (now TIBCO, Palo Alto, CA, USA)) software was used for body weight, condition factor and liver enzyme activity analyses. GraphPad Prism 10 software (GraphPad Software, San Diego, CA, USA) was used for immunity parameter and histopathology index statistical analyses. After checking the normality of the distribution with the Shapiro–Wilk test and establishing the homogeneity of variance, the data were subjected to a parametric ANOVA and a post-hoc test. If the assumptions of normality were not met, the Kruskal–Wallis test and Dunn’s multiple comparisons test were applied. Differences were considered statistically significant at P-value < 0.05.

## Results

**Fig. 1.**
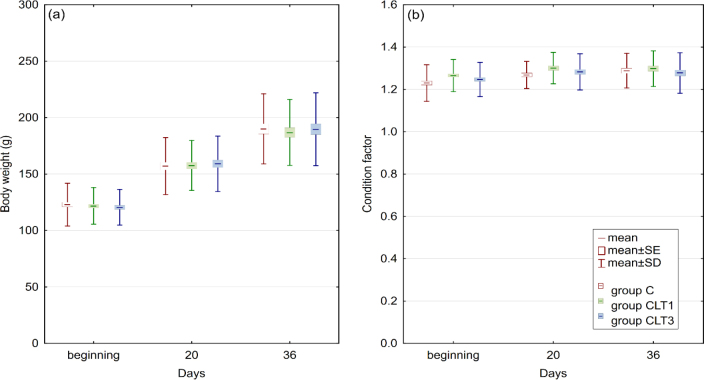
Changes in body weight (a) and condition factor (b) of pikeperch after single and multiple chloramine T exposure (n = 3, P-value ≤ 0.05). C – control group not exposed to chloramine; CLT1 – group exposed once to chloramine on day 0; CLT3 – group exposed three times to chloramine on days 0, 7 and 14

**Fig. 2.**
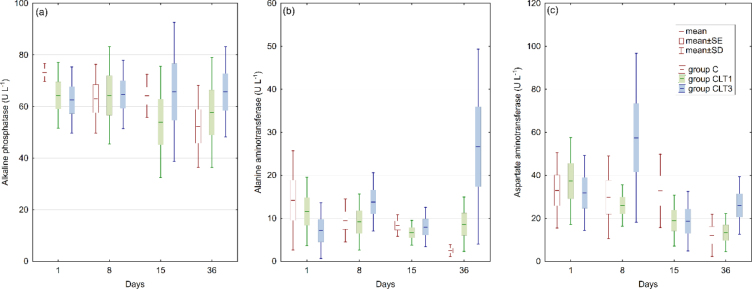
Liver enzyme activity in pikeperch plasma after single and multiple chloramine T exposure (n = 7, P-value ≤ 0.05). C – control group not exposed to chloramine; CLT1 – group exposed once to chloramine on day 0; CLT3 – group exposed three times to chloramine on days 0, 7 and 14

In this study, no significant effect of CLT exposure on fish growth or condition factor was observed between groups (Fig. 1). No changes in liver enzyme activity after CLT exposure were noted (Fig. 2).

**Fig. 3.**
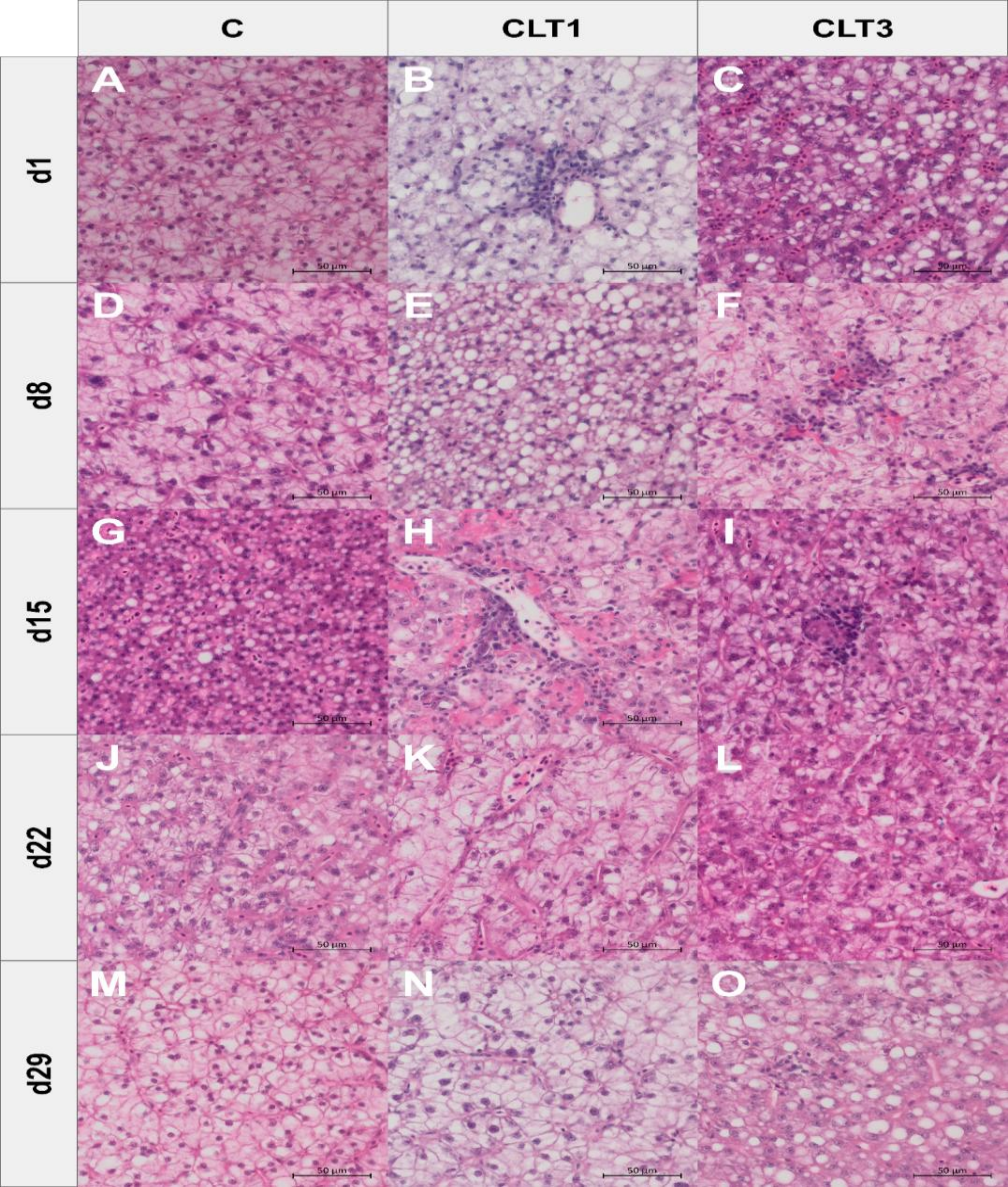
Histopathological changes in pikeperch liver tissue exposed to chloramine in representative sections from different experimental groups. C – control group not exposed to chloramine; CLT1 – group exposed once to chloramine on day 0; CLT3 – group exposed three times to chloramine on days 0, 7 and 14. Differences in coloration are attributed to sexual dimorphism and variations in glycogen accumulation patterns. Images were captured using identical exposure, illumination and white balance and 400× magnification universally. Haematoxylin and eosin staining, scale bar = 50 μm. CLT-associated changes include hepatocyte vacuolisation (B, C, E, G, J, L and O) and mononuclear cell infiltration (B, F, H, I and O)

**Fig. 4.**
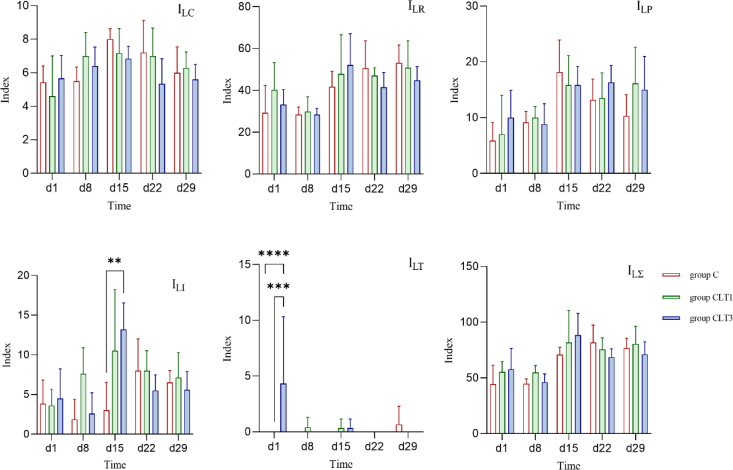
Pikeperch liver response indices after chloramine exposure. I_LC_ – circulatory disturbance index; I_LR_ – regressive change index; I_LP_ – progressive change index; I_LI_ – inflammatory reaction index; I_LT_ – neoplastic change index; I_LΣ_ – total organ index; C – control group not exposed to chloramine; CLT1 – group exposed once to chloramine on day 0; CLT3 – group exposed three times to chloramine on days 0, 7 and 14. Statistically significant differences between groups are marked with asterisks (n = 6, mean ± SD, * – P-value ≤ 0.05; ** – P-value ≤ 0.01; *** – P-value ≤ 0.001; **** – P-value ≤ 0.0001)

Histopathological examination of the liver revealed changes such as hepatocyte vacuolisation and mononuclear cell infiltration in all groups. However, the severity and extent of these lesions, including their distribution within the sample, were most pronounced in the CLT3 group (Fig. 3). Stable values of ILC, ILR and ILP were observed. However, there was an increase in ILI observed on day 15, as well as one in ILT on day 1 in the CLT3 group. There were no changes in IL∑ at any time point (Fig. 4).

The effects of CLT baths on various parameters of non-specific humoral immunity are shown in Table 1. The analysis of the results revealed that lysozyme activity in the serum of pikeperch increased only on day 1 in both experimental groups and on day 22 in the CLT1 group. The other examined parameters of humoral immunity remained unchanged throughout all sampling periods.

**Table 1.** Humoral indicators of non-specific immunity of pikeperch after chloramine T exposure

Group		Day				
		1	8	15	22	29
		Mean ± SD	Mean ± SD	Mean ± SD	Mean ± SD	Mean ± SD
LSM (mg L^−1^)	C	65.63 ± 4.22^a^	69.58 ± 7.67	76.56 ± 5.61	73.75 ± 11.35^a^	77.92 ± 3.16
	CLT1	101.65 ± 6.87^b^	63.34 ± 11.90	75.33 ± 14.59	112.51 ± 4.02^b^	76.41 ± 4.87
	CLT3	94.76 ± 17.79^b^	64.61 ± 10.64	78.91 ± 5.16	84.87 ± 4.38^a^	72.95 ± 4.81
CP (IU)	C	57.06 ± 5.39	48.05 ± 3.48	51.43 ± 4.79	42.59 ± 3.72	57.06 ± 5.39
	CLT1	61.79 ± 5.82	47.59 ± 4.68	51.99 ± 5.92	45.87 ± 2.87	61.97 ± 5.82
	CLT3	60.49 ± 6.89	52.03 ± 9.32	55.25 ± 4.88	46.74 ± 2.72	60.49 ± 6.89
T-P (g L^−1^)	C	43.56 ± 6.15	47.19 ± 5.87	53.36 ± 25.07	47.34 ± 12.66	43.56 ± 6.15
	CLT1	57.79 ± 18.11	41.47 ± 5.91	76.49 ± 43.85	51.57 ± 19.44	57.79 ± 18.11
	CLT3	59.35 ± 13.21	51.99 ± 8.61	64.45 ± 28.35	59.31 ± 14.69	59.35 ± 13.21
γ-G (g L^−1^)	C	7.21 ± 2.06	13.46 ± 1.41	14.63 ± 2.98	15.23 ± 1.93	12.05 ± 3.77
	CLT1	8.80 ± 0.65	13.72 ± 2.52	17.35 ± 2.70	12.96 ± 4.32	9.63 ± 1.72
	CLT3	9.32 ± 2.21	10.69 ± 2.38	16.24 ± 1.72	17.16 ± 2.19	12.95 ± 2.08

1 LSM – lysozyme activity; CP – ceruloplasmin activity; T-P - total protein level; γ-G – gammaglobulin level; C – control group not exposed to chloramine; CLT1 – group exposed once to chloramine on day 0; CLT3 – group exposed three times to chloramine on days 0, 7 and 14. Different letters indicate significant differences, and common letters indicate no significant differences between groups (n = 6, mean ± SD, P-value ≤ 0.05)

Cellular immune parameters are summarised in Table 2. Repeated CLT exposure produced the most significant effects. Respiratory burst activity was notably reduced in the CLT3 group on day 15 compared to both the control and CLT1 groups. Potential killing activity in group CLT3 was also lower on days 15 and 22, whereas on day 29, this group showed a significantly higher PKA than the control. The proliferative responses of T lymphocytes were increased in the CLT3 group compared to the C group on days 8 and 15. On day 22, both CLT1 and CLT3 showed heightened T-cell proliferation relative to C. The proliferation of B cells was significantly increased in CLT3 on days 8 and 15.

**Table 2.** Cellular indicators of non-specific immunity of pikeperch after chloramine T exposure

Group		Day				
		1	8	15	22	29
		SI ± SD	SI ± SD	SI ± SD	SI ± SD	SI ± SD
RBA (SI)	C	1.446 ± 0.32	1.115 ± 0.33	1.002 ± 0.06a	1.042 ± 0.09	1.079 ± 0.23
	CLT1	1.203 ± 0.14	1.072 ± 0.09	1.038 ± 0.12^a^	1.072 ± 0.29	1.131 ± 0.36
	CLT3	1.534 ± 0.40	1.107 ± 0.39	0.773 ± 0.15^b^	1.043 ± 0.18	1.317 ± 0.20
PKA (SI)	C	1.027 ± 0.03	0.818 ± 0.22	0.860 ± 0.07^a^	1.411 ± 0.41^a^	0.719 ± 0.10^a^
	CLT1	0.867 ± 0.07	1.103 ± 0.34	0.741 ± 0.14^ab^	1.159 ± 0.23^ab^	0.803 ± 0.21^ab^
	CLT3	0.942 ± 0.31	0.875 ± 0.33	0.631 ± 0.10^b^	0.798 ± 0.15^b^	0.963 ± 0.12^b^
MTT ConA (SI)	C	0.799 ± 0.09	0.596 ± 0.05^a^	0.754 ± 0.10^a^	0.642 ± 0.06^a^	0.930 ± 0.24
	CLT1	0.664 ± 0.03	0.818 ± 0.09^ab^	0.804 ± 0.06^a^	1.925 ± 0.38^b^	1.000 ± 0.54
	CLT3	0.773 ± 0.13	1.097 ± 0.22^b^	1.744 ± 0.38^b^	1.461 ± 0.18^b^	0.577 ± 0.61
MTT LPS (SI)	C	1.069 ± 0.04	0.970 ± 0.09^a^	0.668 ± 0.16^a^	0.790 ± 0.19^a^	1.127 ± 0.11
	CLT1	1.000 ± 0.11	1.099 ± 0.09^ab^	0.932 ± 0.06^a^	1.374 ± 0.46^b^	1.280 ± 0.34
	CLT3	1.033 ± 0.09	1.192 ± 0.12^b^	1.583 ± 0.59^b^	1.153 ± 0.17^ab^	1.149 ± 0.35

1 RBA – respiratory burst activity; SI – stimulation index; PKA – potential killing activity; MTT Con A – 3-(4,5-dimethyl thiazol-2-yl)-2,5-diphenyltetrazolium bromide concanavalin A assay for proliferative response of T lymphocytes; MTT LPS – 4,5-dimethyl thiazol-2-yl)-2,5-diphenyltetrazolium bromide lipopolysaccharide assay for proliferative response of B lymphocytes. Different letters indicate significant differences, and common letters indicate no significant differences between groups (n = 6, SI ± SD, P-value ≤ 0.05)

## Discussion

In aquaculture, stress significantly affects fish productivity, much like it does in other species. Under stress, the body produces elevated levels of adrenaline and cortisol, promoting catabolism while inhibiting anabolism, leading to slow or stagnant growth. Additionally, stress reduces food intake, absorption and utilisation, forcing fish to expend more energy coping with stressors rather than supporting growth and overall performance (2). Environmental disturbances, such as poor water quality or chemical variations, are key stressors in aquaculture. One such chemical is CLT, which gradually releases aqueous free-chlorine species. While this slow-release mechanism may be effective for certain applications, it can be harmful to aquatic life (22). Research on rainbow trout (*Oncorhynchus mykiss*) revealed that exposure to CLT at 10 mg L^−1^ for one hour twice a week over 11 weeks significantly suppressed growth (24). Similarly, rainbow trout fry subjected to a 20 mg L^−1^ bath once a week also had inhibited growth (17). However, in our study, no significant differences in body weight or condition factor were observed in pikeperch and no mortality was recorded. This suggests that the CLT doses used did not cause toxic effects severe enough to inhibit growth.

Besides growth parameters, liver function is a key indicator of fish health in toxicology studies. It plays a key role in xenobiotic metabolism by processing and eliminating foreign compounds, including drugs, environmental toxins and dietary chemicals (15). Monitoring enzyme activity is a fundamental method for assessing liver health in exposed fish. Among the enzymes involved, ALT and AST are valuable indicators of tissue damage and stress in aquaculture biomonitoring (26). Additionally, ALP serves as a bioindicator of cellular health, hepatocyte function and detoxification processes (4). Liver enzyme measurements provide a sensitive method for detecting inflammation and necrosis, as damaged hepatocytes release these enzymes into the bloodstream (30). Several studies have explored the effects of CLT on liver function. In rainbow trout, brown trout (*Salmo trutta*) and grayling (*Thymallus thymallus*), exposure to 9 g m^−3^ CLT for 20 min three times a day every three days resulted in significant biochemical changes. The rainbow trout exhibited decreased ALT and AST activity but increased lactate dehydrogenase. The brown trout (*Salmo trutta*), however, showed elevated ALT, while the grayling demonstrated an increase in AST (30). Additionally, Boran and Altinok (7) reported an increase in hepatic catalase activity in rainbow trout exposed to therapeutic CLT concentrations, while SOD activity showed the opposite trend. In contrast, our study found no differences in liver enzyme activity. However, histopathological evaluations revealed mild inflammation in the CLT3 group on day 15, suggesting that repeated exposure may cause transient liver tissue changes. Despite this, the overall organ index remained unaffected, indicating no long-term damage. In zebrafish (*Danio rerio*), degenerative liver changes and hepatocyte necrosis were observed after CLT exposure, with lesion severity correlating with exposure concentration (1). Similar results were reached by Gaikowski *et al*. (12) who examined walleye (*Sander vitreum*) and channel catfish (*Ictalurus punctatus*) given 12 consecutive, once-daily, 180-min static immersion baths of 0–80 mg L^−1^ CLT. Histological changes related to exposure did not occur in the liver, but diffused-to-multifocal inflammatory and haemodynamic changes (congestion, leukocyte infiltration, *etc.*) were noted in the spleens of fish. Degenerative changes (erythrocyte necrosis, karyorrhexis or pyknosis) were limited to fish exposed to CLT concentrations higher than recommended.

The fish immune system operates in crucial physiological processes that are highly susceptible to environmental stressors and xenobiotics. As the primary defence against both infectious and non-infectious diseases, the system is particularly sensitive to toxins and chemical agents. Numerous studies have shown that the nature, intensity and duration of exposure to these foreign substances critically influence immune responses. Additionally, factors such as life stage, sex and species further modulate immunocompetence and the capacity to cope with immunotoxic stressors. Interestingly, fish often demonstrate enhanced immune functions following acute stress, such as increased levels of lysozymes, cytotoxic cells and phagocytes. This mobilisation of the immune system appears to be an adaptive mechanism for protection against potential future threats. Our results in pikeperch indicated that serum lysozyme activity increased after the first CLT exposure. This may suggest that the fish were experiencing mobilising stress, characterised by a temporary physiological response aimed at adapting to a sudden environmental change. Yildiz *et al*. (33) reported that CLT at a concentration of 5 ppm for 3 h had no effect on plasma lysozyme levels in rainbow trout. They also did not notice any changes in ceruloplasmin and total protein, which is consistent with our results. In contrast, chronic or repeated stress is often associated with detrimental effects, including immune suppression (13, 20). For instance, studies on common carp (*Cyprinus carpio*) exposed to elevated CLT concentrations (20, 100 and 200 mg L^−1^) for 15 days revealed a significant decrease in serum lysozyme activity, followed by partial recovery during the post-exposure period. A substantial reduction in total immunoglobulin levels at 200 mg L^−1^ further highlights the immunosuppressive potential of high-dose CLT exposure (9).The cellular immunity parameters revealed significant treatment-dependent changes in phagocytic function and lymphocyte proliferation (Table 2). Stressful stimuli typically induce redistribution of immune cells, with mobilisation of monocytes and granulocytes from lymphoid organs into circulation, while simultaneously modulating cellular effector functions (31). However, activation and deactivation of the defence system are determined by the duration and the intensity of the stress experienced by the organism (25). In our study, a single CLT exposure did not elicit significant changes in cellular immune parameters; however, repeated CLT exposure was associated with a pronounced reduction in phagocyte respiratory burst activity on day 15. A parallel decline in PKA observed on days 15 and 22 indicated that repeated CLT treatments temporarily impaired phagocyte-mediated microbial killing. Such suppression of macrophage/phagocyte function under repeated or chronic stress has been documented in teleosts and may be mediated by neuroendocrine pathways, because early stress responses involving the autonomic nervous system can rapidly depress phagocytic activity (16). Importantly, this suppression was reversible. Killing activity increased markedly approximately two weeks after the final exposure, indicating that phagocyte function recovered following cessation of chemical stress. Comparable transient suppression and recovery of phagocyte function after CLT exposure were reported by Dar *et al*. (9) in common carp, reinforcing the idea of a reversible, exposure-dependent immunomodulation. We detected increased proliferation of both T and B lymphocytes in the CLT3 group. This shift from suppressed innate phagocytic functions toward enhanced adaptive cellular responses may reflect a compensatory activation of the lymphocyte compartment in reaction to tissue inflammation or altered antigenic exposure following repeated CLT baths. Histopathological evidence of hepatic inflammation and previously reported gill lesions in pikeperch after multiple CLT treatments (27) support the notion that tissue damage or increased antigen presentation could stimulate lymphocyte proliferation. Similar changes were noted in channel catfish and walleye after CLT exposure (22, 24). Taken together, these results suggest that repeated CLT applications can temporarily compromise innate antimicrobial defences while promoting adaptive immune activation. Our data support avoiding repeated CLT baths at short intervals and recommend allowing recovery intervals and monitoring immune markers and water quality closely to minimise windows of transient immunosuppression. The net effect on disease susceptibility will depend on timing. Transient phagocyte suppression during or shortly after treatment could increase vulnerability to opportunistic pathogens, whereas subsequent lymphocyte activation may aid longer-term immune protection. To clarify mechanisms and functional consequences, future studies should include time-series measurements of stress indicators. The observed increase in B- and T-lymphocyte proliferation on day 22 in the CLT1 group and the elevated LSM activity indicate that these changes may be driven by distinct underlying factors that are likely independent of the experimental conditions. Despite our efforts, we have not been able to conclusively identify the precise cause of this increase, and it remains unclear based on the information available at this time.

It is crucial to recognise that the use of chemotherapeutants in RAS bath treatments does more than target harmful pathogens – it also disrupts beneficial microbial strains essential for the biofilter’s function. In RAS, these treatments may temporarily or persistently reduce nitrifying bacteria, which are essential for efficient nitrification and a stable nitrogen cycle (3). Therefore, chemical interventions should be carefully managed to maintain system stability. Noble and Summerfelt (17) reported that CLT at concentrations up to 12 mg L^−1^ did not impair biofilter performance or nitrification efficiency. In our study, a brief decrease in nitrification occurred after exposure to CLT, but total ammonia nitrogen (TAN) and nitrite (NO_2_^–^) levels stabilised within 2–3 days. It should be emphasised, however, that the level of ammonia and nitrites during the research was within the range of safe values for pikeperch (10) at all times. Occasional use of CLT at the tested concentration is appropriate for well-managed RAS if dosing is conservative and nitrification is closely monitored.

## Conclusion

The present study demonstrates that CLT can be safely used in pikeperch at the tested dose and over the tested exposure time. Used this way, the compound exerts no negative effects on growth or survival under controlled recirculating aquaculture system conditions. However, changes in immune parameters and liver inflammation, especially after repeated exposures, show how sensitive the pikeperch immune system is to repeated chemical stress. These findings highlight the importance of careful dose selection and timing for CLT treatments in aquaculture. This is especially important for species such as pikeperch, which are new introductions to intensive farming. Acute CLT exposure may give a brief, adaptive boost to immune function like lysozyme activity, but chronic or excessive use could risk immunosuppression and disrupt physiology. Further research should refine species-specific treatment, determine long-term effects and optimise the balance between treatment efficacy and fish welfare. Finally, because we did not measure biofilter microbes or nitrification rates directly, future studies should include molecular and functional tests.
